# End‐Stage Renal Disease Patient on Hemodialysis With Baclofen‐Related Neurotoxicity and Pancreatitis

**DOI:** 10.1155/crin/5482934

**Published:** 2026-04-07

**Authors:** Abdulaziz Alroshodi

**Affiliations:** ^1^ Department of Medicine, College of Medicine, Qassim University, Buraidah, Saudi Arabia, qu.edu.sa

**Keywords:** baclofen, end-stage renal disease, hemodialysis, neurotoxicity, pancreatitis, toxicity

## Abstract

**Background:**

Baclofen is a centrally acting muscle relaxant used orally and intrathecally for the management of spasticity, muscular spasms, and refractory hiccups. Baclofen is predominantly absorbed via the gastrointestinal tract, with over 80% excreted via the kidneys; hence, individuals with end‐stage renal disease (ESRD) are at a heightened risk for baclofen toxicity. Baclofen can penetrate the blood–brain barrier, resulting in neurotoxicity. The potential of baclofen to induce acute pancreatitis is inadequately comprehended, with a limited number of instances documented in the medical literature. This report details an uncommon instance of an ESRD patient undergoing hemodialysis who had both baclofen‐induced neurotoxicity and pancreatitis.

**Case Presentation:**

We present a case of a 55‐year‐old woman who is known to have ESRD; she is currently on hemodialysis three times a week. After only two baclofen doses (10 mg, 12 h apart), given for chronic cervical pain, the patient developed a profound decrease in her level of consciousness (LOC). The next day, when her LOC had improved after dialysis, she reported nausea, vomiting, and epigastric abdominal pain as well. Her initial laboratory report showed an elevated amylase level. So, the assessment was a baclofen neurotoxicity in an ESRD patient, which was accompanied by mild–moderate pancreatitis after only two doses. Since baclofen is highly dialyzable, she was commenced on daily conventional hemodialysis using a high‐flux dialyzer for three consecutive days. She did not require continuous dialysis. Her LOC improved dramatically after the second session, and she returned to her baseline just after the third session. However, she reported headache and hallucinations after. Pancreatitis symptoms improved with supportive measures later.

**Conclusion:**

The case highlights the need for increased awareness among healthcare providers about the potential risks of baclofen use, particularly in patients with ESRD. Even a small dosing can cause neurotoxicity. This is a very rare case of baclofen neurotoxicity accompanied by pancreatitis, who did respond very well to conventional hemodialysis. Patients with decreased renal function should avoid using baclofen, as its accumulation depends on renal excretion capacity.

## 1. Introduction

Baclofen, β‐4‐chlorophenyl gamma‐aminobutyric acid (GABA), is a natural derivative of the neurotransmitter GABA [1]. It is regarded as an active agonist for GABAB receptors. It is a centrally acting muscle relaxant used orally and intrathecally for the management of spasticity, muscular spasms, and refractory hiccups [2]. It has been improperly utilized as a “recreational drug” by teenagers [3]. It successfully alleviates symptoms including flexor spasm, clonus, and discomfort [4].

Baclofen is predominantly absorbed through the gastrointestinal system, with over 80% eliminated by the kidneys. This elucidates why those with diminished renal function are at an elevated risk of intoxication compared to those with normal renal function [5]. The half‐life of baclofen varies from 4.5 to 6.8 h. The half‐life is prolonged in individuals with chronic kidney disease (CKD), especially in those with end‐stage renal disease (ESRD), where the majority of baclofen toxicity cases are documented among dialysis patients [6]. Baclofen penetrates the blood–brain barrier slowly, resulting in limited access to the central nervous system (CNS). Nevertheless, its longer half‐life increases CNS penetration, potentially leading to neurotoxicity [5, 7].

Acute pancreatitis is a condition characterized by inflammation of the pancreas. The usual presentation is epigastric pain, which may extend to the back, accompanied by elevated pancreatic enzymes (amylase and lipase). Drug‐induced pancreatitis is an uncommon yet notable etiology of acute pancreatitis, attributable to a range of pharmaceuticals [8]. The potential of baclofen to induce acute pancreatitis is little comprehended, with a limited number of instances documented in the medical literature [9]. To the best of our knowledge, there were no case reports showing pancreatitis in ESRD patients on hemodialysis with baclofen‐related neurotoxicity. Herein, we presented the first case of an ESRD patient on hemodialysis with baclofen‐related both neurotoxicity and pancreatitis.

## 2. Case Presentation

This is a 55‐year‐old woman who is known to have ESRD due to an undetermined etiology, currently on hemodialysis three times a week (Sun–Tue–Thu) using a well‐functioning left brachiocephalic AVF. Also, she is known to have severe hyperparathyroidism status postparathyroidectomy in 2022. She has an excellent functional and cognitive status as a baseline. She is adherent to her dialysis sessions and is doing fine on hemodialysis.

She presented on Wednesday night (off‐dialysis day) with an altered level of consciousness (LOC) that had been worsening over the last 24 h. She ingested two tablets of baclofen 10 mg, 12 h apart (total of 20 mg in 12 h) for chronic cervical pain. She already took another 10 mg on Monday night, just prior to her Tuesday session, but she did not develop any symptoms, as she had dialysis just after the ingestion. According to her family, her LOC had worsened to the degree that she was not responsive, for which she was brought to the emergency.

There was no seizure, abnormal limb position, dysphagia, dysarthria, facial asymmetry, urinary or fecal incontinence, fever, photophobia, or phonophobia. The family denied trauma, contact with sick patients, fever, and neck stiffness apart from chronic pain that she has had for years, which is related to cervical vertebrae problem. She does not drink or smoke and did not take other new drugs.

The next day, when her LOC had improved after one dialysis session, she reported nausea, vomiting, and epigastric abdominal pain.

On examination in the emergency room (ER), she looked ill and obtunded, semiconscious yet not oriented and not able to obey simple commands. Her Glasgow Coma Scale (GCS) was 8 (E2 V2 M4). Her vital signs were normal (BP 150/74 mmHg, HR 82 BPM, RR 18 BPM, O_2_ 96%, and temperature 36.2°C). She was saturating well on room air (RA). Her pupils were equal and reactive to light. There was no facial asymmetry or gross limb abnormality. Deep tendon reflexes were intact; however, the tone was increased. Her abdomen was soft and lax with epigastric tenderness. Her other systems’ examinations were unremarkable.

Her initial laboratory investigations were normal, including complete blood count (CBC), electrolytes, liver function tests, blood glucose, viral serology, creatine phosphokinase (CPK), and CPK‐MB. Her creatinine and blood urea nitrogen (BUN) were within the range of ESRD patients. However, her amylase level was high at 605 U/L (normal range: 28–100). Lipase and baclofen levels were not available. Her triglyceride and calcium levels were within the normal range.

Her ECG showed a normal sinus rhythm with no ST, QRS, or QTc changes. Computed tomography (CT) of the brain did not show any acute intracranial insult. Ultrasound (US) of the abdomen showed no abnormalities apart from kidney changes compatible with ESRD. The pancreas was masked by gases. There were no gallstones.

So, the initial assessment was baclofen‐related neurotoxicity. Hyperamylasemia was expected in dialysis patients; however, with that high level, in addition to the typical symptoms, the diagnosis of mild–moderate pancreatitis was made.

She was commenced on daily hemodialysis for three consecutive days, 4 h each, using a high‐flux, large‐sized dialyzer with the maximum blood flow. She started to improve immediately after the 1st session when her GCS had improved (Table 1). Her dramatic improvement was after the 2nd session. She did not require any form of continuous dialysis. After the third hemodialysis session, she returned to her normal baseline. However, she continued to report some headaches. During her hospital stay, she developed transient auditory and visual hallucinations.

**TABLE 1:** GCS improvement.

In ER	After the 1st dialysis session	After the 2nd dialysis session Significant improvement	After the 3rd dialysis session
8 (E2 V2 M4)	11 (E3 V4 M4)	14 (E4 V4 M6)	15 (E4 V5 M6)

Her pancreatitis symptoms had improved over the next few days with intravenous (IV) fluids and other supportive measures. CT abdomen was deferred due to significant clinical improvement to avoid unnecessary contrast and radiation exposure.

## 3. Discussion and Conclusion

Baclofen is utilized for the management of spasticity, muscular spasms, and persistent hiccups. The recommended dosage for spasticity therapy is 5 mg three times a day, with a maximum daily dose of 80 mg. For hiccups, the dosage is 10–20 mg three times daily [10]. Eighty percent of the medicine is excreted unaltered in urine, while the liver metabolizes the remainder. The aging process, uremia, and associated CNS disorders elevate the likelihood of toxic consequences [11]. The drug has a half‐life of 4.5–6.8 h in healthy individuals, which is extended in patients with renal failure; hence, the majority of baclofen toxicity cases are documented in dialysis cases [10, 11]. Baclofen is rapidly absorbed, with a bioavailability of 70% to 85%, and achieves peak plasma concentrations within 2 to 3 h. Its absorption is dose‐dependent, and the drug has a distribution volume of 0.7 L/kg. It exhibits high water solubility, and its protein‐binding rate is 30%. Due to a relatively short half‐life, baclofen necessitates frequent dosing. It is primarily eliminated through renal excretion [12]. Since baclofen elimination is significantly reliant on renal clearance, individuals with impaired kidney function are at elevated risk of baclofen toxicity.

Initial case reports indicated that individuals with ESRD experienced a changed mental state following the administration of minimal dosages of baclofen for brief durations; however, pancreatitis in ESRD patients on hemodialysis with baclofen toxicity has never been reported. Herein, we presented the first case of an ESRD patient on hemodialysis with baclofen‐related neurotoxicity and pancreatitis.

Approximately 50 cases of baclofen toxicity have been documented in individuals with CKD, primarily those undergoing dialysis [11, 13]. An analysis of 41 reported instances of baclofen toxicity indicated that affected individuals were predominantly elderly and largely reliant on dialysis [11]. Neurotoxicity has been seen in individuals using 5–60 mg of baclofen daily (median dosage 20 mg/day) as early as 2‐3 days and up to 16 weeks postinitiation of the medication [10]. This aligns with our findings, as the ESRD patient exhibited symptoms within the first 24 h after receiving the initial dosages and subsequently underwent hemodialysis, which resulted in symptom improvement.

Khazneh et al. [1] documented encephalopathy in a patient 12 h postingestion of a single 25 mg baclofen pill, necessitating five dialysis treatments for complete recovery. Radhakrishnan et al. [14] documented a dialysis patient who had toxicity after a single administration of 10 mg. Hadjiyannacos et al. [15] proposed that a dosage of 2.5 mg baclofen may be safer for dialysis patients than the 5 mg dose. Regarding our patient, she developed baclofen‐related neurotoxicity symptoms after two doses of baclofen, 10 mg each, and 12 h apart. So, baclofen is dangerous in dialysis population regardless of the dose.

Baclofen toxicity often manifests as a range of altered mental status, from sleepiness to coma [10, 11]. Additional side effects include CNS inhibition, lethargy, and nausea [10, 11, 13]. Hallucinations, seizures, and delirium occur seldom. In this case, after the first two doses, the patient experienced a profound decrease in her LOC. However, she had no symptoms when she took the dose just before the hemodialysis session (Monday night dose), as she was dialyzed immediately for her conventional session. Dialysis most likely did remove her Monday dose, so she had no symptoms. Baclofen is 79% dialyzable [16].

Drug‐induced pancreatitis is an uncommon illness, especially when combined with drugs not previously connected to pancreatic inflammation. This instance suggests a possible correlation between baclofen administration and pancreatitis, given the patient’s symptoms after initiation and the absence of any discernible explanations. Physicians must acknowledge the potential of baclofen to induce inflammation when giving it and consider it as a differential diagnosis in instances of unexplained acute pancreatitis [9]. Nonetheless, direct causality remains ambiguous, necessitating more study to clarify the possible association between baclofen and pancreatic inflammation [9]. Our patient was diagnosed with mild–moderate pancreatitis, meeting the diagnostic criteria, including epigastric pain and elevated amylase level, keeping in mind the limitation of the amylase level in ESRD patients. Her US excluded gallstones. Her triglyceride and calcium levels were within the normal range. Baclofen‐related pancreatitis has been reported in a case report published by Nassif et al. [9]. However, the patient in the latter case was a nondialysis CKD patient. To the best of our knowledge, there have been no previous case reports of baclofen‐related pancreatitis in ESRD patients. Also, we are the 1st report with both neurotoxicity and pancreatitis.

The cornerstone of therapy is supportive care and vigilant monitoring for any respiratory or hemodynamic compromise [5]. Hemodialysis is a highly effective therapeutic approach as baclofen is highly dialyzable [17]. It reduces the half‐life of baclofen in patients with ESRD from 15 to 2 h [17]. Some recorded cases exhibited significant improvement after a single session, as it is believed to expunge baclofen from the body in a manner similar to normal renal function. However, some patients required up to five sessions for full recovery. Our patient was commenced on daily hemodialysis for three consecutive days, improving partially after the first session, and significantly after the second session. After the third session, she returned to normal but continued to report headaches and hallucinations. Pancreatitis symptoms improved with IV fluids and supportive measures. Though continuous renal replacement therapy (CRRT) appears to be an effective alternative for hemodynamically unstable individuals [18], our patient did not require it.

In conclusion, this case highlights the necessity for increased vigilance among healthcare practitioners concerning the possible hazards of baclofen usage, particularly in ESRD patients, where even a small dose may induce neurotoxicity. Moreover, this case represents an uncommon instance of baclofen neurotoxicity compounded with pancreatitis. Upon diagnosis and the prompt commencement of hemodialysis, complete reversibility without sequelae is achievable. Increased efforts are necessary to educate healthcare personnel about the potential dangers associated with baclofen usage in cases of ESRD. We advise against using baclofen in patients with impaired renal function, including those with moderate chronic renal failure, due to the cumulative buildup in the CNS, which is contingent upon renal excretion capability. Consequently, we strongly advise exploring alternative options.

**Nomenclature**
ESRD: End‐stage renal disease
IV: Intravenous
US: Ultrasound
GABA: Gamma‐aminobutyric acid
CKD: Chronic kidney disease
LOC: Level of consciousness
CNS: Central nervous system
GCS: Glasgow Coma Scale
ER: Emergency room
BP: Blood pressure
RR: Respiratory rate
BPM: Beat per minute
RA: Right atrium
CBC: Complete blood count
BUN: Blood urea nitrogen
CPK: Creatine phosphokinase
CT: Computed tomography
CAPD: Continuous ambulatory peritoneal dialysis
CRRT: Continuous renal replacement therapy

## Author Contributions

Dr. Abdulaziz Alroshodi was the sole author and contributor to the conception, patient data gathering, discussion writing, and manuscript preparation.

## Funding

No funding was received for the preparation of this case report.

## Ethics Statement

Ethical approval was not required for this single‐patient case report, as it does not meet the definition of human subject research according to institutional and national guidelines. The report was prepared in accordance with the ethical standards of our institution and with the principles of the Declaration of Helsinki.

The authors confirm that they have acquired all necessary patient consent forms, including written informed consent from the patient herself, to share and publish the patient’s clinical information. The patient acknowledged that her name and initials will not be disclosed, and every effort will be made to protect her identity, although complete anonymity cannot be assured.

## Consent

Please see the Ethics Statement.

## Conflicts of Interest

The author declares no conflicts of interest.

## Data Availability

Data sharing is not applicable as no datasets were generated or analyzed in this single case report.
